# Health professionals’ views and experiences of the Australian moratorium on genetic testing and life insurance: A qualitative study

**DOI:** 10.1038/s41431-022-01150-6

**Published:** 2022-07-28

**Authors:** Grace Dowling, Jane Tiller, Aideen McInerney-Leo, Andrea Belcher, Casey Haining, Kristine Barlow-Stewart, Tiffany Boughtwood, Penny Gleeson, Martin B. Delatycki, Ingrid Winship, Margaret Otlowski, Chris Jacobs, Louise Keogh, Paul Lacaze

**Affiliations:** 1 Public Health Genomics, School of Public Health and Preventive Medicine, Monash University, Melbourne, Australia; 2 Murdoch Children’s Research Institute, Parkville, Australia; 3 The University of Queensland Diamantina Institute, University of Queensland, Dermatology Research Centre, Brisbane, Australia; 4 Faculty of Medicine, University of Queensland, Brisbane, Australia; 5 Australian Genomics, Melbourne, Australia; 6 Centre for Health Equity, Melbourne School of Population and Global Health, The University of Melbourne, Melbourne, Australia; 7 Sydney Medical School, University of Sydney, Sydney, Australia; 8 Deakin Law School, Melbourne, Australia; 9 Victorian Clinical Genetics Services, Parkville, Australia; 10 Department of Medicine, The University of Melbourne, Melbourne, Australia; 11 Genomic Medicine and Family Cancer Clinic, Royal Melbourne Hospital, Parkville, Australia; 12 Faculty of Law and Centre for Law and Genetics, University of Tasmania, Hobart, Australia; 13 Graduate School of Health, University of Technology Sydney, Sydney, Australia

**Keywords:** Genetic counselling, Genetics, Public health, Ethics

## Abstract

Australian life insurance companies can legally use genetic test results in underwriting, which can lead to genetic discrimination. In 2019, the Financial Services Council (Australian life insurance industry governing body) introduced a partial moratorium restricting the use of genetic testing in underwriting policies ≤ $500,000 (active 2019–2024). Health professionals (HPs), especially clinical geneticists and genetic counsellors, often discuss the implications of genetic testing with patients, and provide critical insights into the effectiveness of the moratorium. Using a sequential explanatory mixed methods design, we interviewed 23 Australian HPs, who regularly discuss genetic testing with patients and had previously completed an online survey about genetic testing and life insurance. Interviews explored views and experiences about the moratorium, and regulation, in greater depth. Interview transcripts were analysed using thematic analysis. Two key themes emerged from views expressed by HPs during interviews (about matters reported to or observed by them): 1) benefits of the moratorium, and 2) concerns about the moratorium. While HPs reported that the moratorium reassures some consumers, concerns include industry self-regulation, uncertainty created by the temporary time period, and the inadequacy of the moratorium’s financial limits for patients’ financial needs. Although a minority of HPs felt the current industry self-regulated moratorium is an adequate solution to genetic discrimination, the vast majority (19/23) expressed concern with industry self-regulation and most felt government regulation is required to adequately protect consumers. HPs in Australia are concerned about the adequacy of the FSC moratorium with regards to consumer protections, and suggest government regulation is required.

## Introduction

Genetic testing can help identify individuals’ risk of developing future disease, including some cancers [1], and can effect positive health outcomes through prevention or early detection and treatment where available. In Australia, genetic test results can also lead to genetic discrimination in life insurance, including increased premiums or denial of cover on the basis of genetic test results [2]. Fear of life insurance discrimination has been shown to deter individuals from undergoing predictive genetic testing [3] and participating in genomic research [4].

Debate exists regarding whether the use of genetic test results by life insurers should be restricted [5]. Some contend that the use of genetic information is a necessary and accepted principle of life insurance underwriting. Others, including many governments internationally [6, 7], have accepted that curtailment of this is necessary for the protection of certain human rights, including those protected by Article 6 of the United Nations Universal Declaration on the Human Genome and Human Rights (unanimously adopted by 77 countries, including Australia), and Article 25 of the UN Convention on the Rights of Persons with Disabilities (which Australia has confirmed) [8]. Many countries, including the United Kingdom, Canada, and many European nations, have restricted or banned the use of genetic test results in life insurance underwriting [6, 7, 9]. Private life insurance in those countries has not become unviable so far, suggesting that this debate is not determined, but rather an issue on which there are various points of view.

In Australia, under the *Disability Discrimination Act 1992* (Cth) [10], insurance companies can legally use an individual’s genetic status to discriminate against them in underwriting risk-rated insurance, if the company can justify its reasoning with actuarial or statistical data [1]. This allowance does not apply to health insurance, which must be community-rated under separate legislation [11] and is thus protected from genetic discrimination. Risk-rated insurance cover underwritten by life insurance companies in Australia includes life (death) cover, income protection, total and permanent disability, and critical illness/trauma cover.

Australian life insurance companies are self-regulated by the industry governing body, the Financial Services Council (FSC). The FSC self-regulates its own access to, and use of, genetic test results through mandatory practice standards, without government oversight [12]. Despite previous efforts [13, 14], the Australian government has not taken steps to limit insurance companies’ use of genetic test results. Following recommendations from a Parliamentary Joint Committee into the life insurance industry that this practice should be banned [13], however, the FSC introduced an industry-led, partial moratorium (ban) on use of genetic test results for life insurance products applied for after July 1 2019 [15]. The FSC moratorium is not a complete ban – protection is only offered for policies ≤ $500,000 for life (death) cover,  ≤ $4000/month for income protection, ≤ $500,000 for total and permanent disability, and ≤ $200,000 for critical illness/trauma cover. The self-regulated moratorium will expire in 2024 unless renewed, and is not legally enforceable nor subject to government oversight.

In recognition of the importance of this issue, the Australian government has funded a three-year project to monitor the effectiveness of the FSC moratorium: the Australian Genetics and Life Insurance Moratorium: Monitoring the Effectiveness and Response (A-GLIMMER) [16]. The project is a national study, collecting views and evidence from multiple stakeholders (health professionals, consumers, researchers/research participants, and the financial services industry) [17].

Health professionals (HPs), including clinical geneticists and genetic counsellors, play an essential role in assisting patients with making informed choices about genetic testing [18]. HPs must, where relevant, discuss the implications of genetic testing on life insurance, as required by the Australian professional guidelines for genetic counselling [19]. There is little literature regarding HPs’ views and experiences regarding the current FSC moratorium. Understanding these views is an important component for informing its future appropriateness. In this study, we interviewed Australian HPs who had previously responded to an online survey about the moratorium [20], to further explore their views and experiences, adopting a sequential explanatory mixed methods design. The research question addressed was “what are the views and experiences of Australian healthcare professionals regarding the genetics and insurance moratorium?”.

## Methods

This study forms part of the A-GLIMMER project [21]. The first element of this study consisted of an online survey distributed to HPs in 2020, to gather evidence regarding their views and experiences of the moratorium. The results of that survey (*n* = 166) have been published [22]. Here, we undertook follow-up interviews with survey participants who agreed to be contacted in order to expand on and explore the quantitative responses. The interviews allowed for a greater in-depth understanding of individual participants’ views and experiences.

Genetic testing can occur in different contexts, including research, clinical testing, and direct-to-consumer testing, conducted online without the involvement of a health professional. In a clinical context, health professionals facilitate both diagnostic and predictive testing. Given disease diagnoses can be used by underwriters in any event, predictive genetic test results are more relevant for discussions about the impact of life insurance underwriting. Questions in the survey were framed in the context of unaffected adult patients accessing predictive genetic testing in a clinical context.

### Recruitment

Recruitment for the online survey has been described previously [22]. Individuals were eligible for the survey if they were qualified HPs working in Australia who discuss genetic testing with patients. The majority of respondents (73%) were clinical geneticists/genetics fellows and genetic counsellors, with a minority representation from other, non-genetics HPs. At the conclusion of the online survey, participants were asked whether they consented to be contacted for a follow-up interview. No contact details were collected from participants who preferred to remain anonymous. All HPs who consented were contacted via email, approximately 10 months after their initial survey completion, to invite their participation in a follow-up interview.

### Interviews

Semi-structured interviews taking up to 30 min were held by teleconference and carried out by GD and CH between January and April 2021. Participants consented to audio recording and were advised that the recording would be de-identified and transcribed for analysis and publication. The interview schedule (Supplementary File S1) was designed to explore the responses given in the online survey and was tailored to each interview participant, using their survey responses as a starting point. The schedule was developed iteratively –new topics that arose regularly in interviews were incorporated for future interviews.

### Analysis

The audio files were de-identified and transcribed verbatim to allow for thematic analysis. Inductive thematic analysis [23] involves familiarisation with the data, followed by identification of themes in order to determine patterns of meaning in the data. This enabled the research team to present the collective meanings and experiences from the data set [24, 25]. Five transcripts were read by GD to develop an initial coding framework. These five transcripts and the coding framework were reviewed by CJ to confirm full capture of the main themes present in the data. One full transcript was independently coded by CJ to ensure coding consistency. The coding framework was used to determine when the main themes were saturated and no new themes were emerging during the interviews. Once data collection was completed, all transcripts were read by GD and the coding framework revised to incorporate all data. GD, JT and LK collaboratively refined the final coding framework to capture the main themes (Supplementary File S2). The coding of transcripts was performed by GD, and then each code was further analysed collaboratively by GD, JT and LK.

## Results

### Sample

**Fig. 1:**
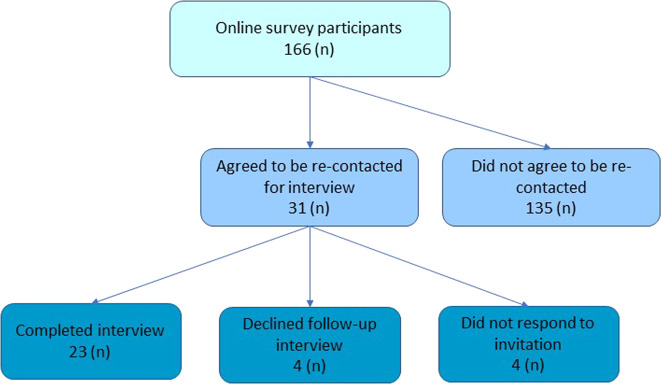
Recruitment outcomes for qualitative interviews.

Thirty-one survey participants agreed to be contacted and were invited for follow-up interviews – of these, four declined to participate and four did not respond to the invitation. Twenty-three participants took part in an interview (Fig. 1). Data saturation occurred at interview 17, and the final six interviews were conducted to confirm saturation on key themes.

The demographics for the 23 interviewed participants are set out in Table 1. The sample had a reasonable spread of sex (48% males), years of experience (43% with at least 15 years’ experience) and location within Australia.

**Table 1:** Participant Demographics.

	Number of participants interviewed *n* = 23	
	*n* = 23	*n*(%)
**Gender**		
Male	11	48%
Female	12	52%
Profession		
Associate Genetic Counsellor	7	30%
Certified Genetic Counsellor*	7	30%
Clinical Geneticist	5	22%
Other^**^	4	18%
**Years of experience**		
0–5 years	8	35%
6–10 years	3	13%
11–15 years	2	9%
15–20 years	3	13%
> 20 years	7	30%
**State**		
Victoria	5	22%
New South Wales	9	39%
Queensland	3	13%
Northern Territory	0	0%
South Australia	1	4%
Western Australia	3	13%
Tasmania	2	9%

*In Australia, qualified genetic counsellors are titled “Associate” until they have completed a certification pathway, after which time they are titled “Certified”.

**Other included: Genetic pathologist, cardiologist, metabolic clinician, chemical pathologist.

Interviews explored the views and experiences of Australian HPs regarding the genetics and insurance moratorium. Many HPs reflected on how the moratorium has been received by their patients, and the opinions they have formed, based on their experiences as HPs and their interactions with patients. Table 2 sets out the main themes and subthemes identified, which are discussed in more detail below.

**Table 2:** Main themes identified through thematic analysis.

Theme	Subtheme
The benefits of the moratorium	Alleviating patient concerns
	Removing perceived barriers for patients
Concerns about the moratorium	Self-regulation by the insurance industry
	Impact of the financial limits
	The uncertainty created by the moratorium’s temporary nature

### Theme 1: The benefits of the moratorium

A number of HPs mentioned some patients telling them that the moratorium provided some reassurance, and made it easier for them to have genetic testing without worrying about implications for their life insurance.

#### Alleviating patient concerns

Several interviewed HPs expressed a view that the moratorium is a step in the right direction and provides benefits for some patients.

*“Look, I think [the moratorium is] a step in the right direction … patients don’t have to even, for low level insurance, disclose genetic test results.”* (ID 13, certified genetic counsellor, 6-10 years’ experience)

HPs also reported comments from patients that the moratorium had reassured them about possible adverse insurance outcomes for their family members, if they choose to have genetic testing.

*“People are more comfortable with any form of genetic testing knowing that there’s a moratorium. Both knowing it for themselves and knowing that if they have it, if something’s found, it won’t impact on their family members.”* (ID 8, certified genetic counsellor, 0-5 years’ experience)

According to some HPs, a handful of patients expressed concern about the implications of genetic testing on their children, including impacting their eligibility to access insurance products if there is a known family genetic condition. HPs reported that the moratorium helped to alleviate this worry for some patients.

#### Removing perceived barriers for patients

HPs reported that with the introduction of the moratorium, some patients felt more secure, as they would be able to access some life insurance cover, which may not have been possible pre-moratorium.

*“I have had positive feedback from patients who have been relieved that if they go ahead with the genetic testing, they can still get a reasonable amount of life insurance cover.” (ID 11, certified genetic counsellor, 15-20 years’ experience)*

Some HPs also commented that not needing to have insurance in place prior to genetic testing removed pressure from some patients, who may otherwise have delayed or declined genetic testing.

*“Now that the moratorium is here it just takes away that added stress of, do I need to get [life insurance] sorted out before I have a genetic test?”* (ID 10, certified genetic counsellor, 11–15 years’ experience)

### Theme 2: Concerns about the moratorium

#### Self-regulation by the insurance industry

The vast majority of interviewed HPs (19/23) expressed concerns about the FSC’s self-regulation of the industry’s adherence to the moratorium, making it the most frequently expressed concern. HPs viewed self-regulation by the insurance industry as inadequate, due to a lack of trust in life insurance companies to abide by the moratorium without any government regulation.

*“I don’t have much trust in the insurance industry as a whole so I guess anything to regulate* [the industry] *that’s from an external body* [would be better] *- government would be the best, because that is our structure of the law… to keep things in check and make it fairer for people.”* (ID 4, associate genetic counsellor, 0–5 years’ experience)

Concerns from HPs included the potential for non-compliance with the moratorium by insurance companies due to lack of government regulation, and lack of penalties for non-compliance. HPs also described their distrust in insurance companies to self-regulate properly because their commercial interests are in direct conflict with patient interests.

*“I think there should be somebody overseeing it rather than just the life insurance companies. It doesn’t make sense because they are – I mean they have a commercial interest in what they’re doing so why should we trust them to do the right thing really?” (ID 10, certified genetic counsellor, 11*–*15 years’ experience)*

Many HPs felt that insurers’ use of genetic information should be regulated by the government through legislation. HPs expressed a view that legislation would hold insurance companies accountable to limitations on their use of patients’ genetic information, helping to ensuring fair treatment of all patients applying for insurance.

*“This is self-monitored, there is no set legislative regulations that the insurance companies, by the law, have to abide by. Or there is no check, per se, on it, and having legislation would make that happen. There is a bit more responsibility.” (ID 1, associate genetic counsellor, 0*–*5 years’ experience)*

*“If it’s law*, [insurance companies] *have to follow it and then if they choose not to, then there are the repercussions of that. They can be liable to criminal charges, I guess. So, yeah, I like that idea in that it holds them accountable for their actions.”* (ID 13, certified genetic counsellor, 6–10 years’ experience)

A minority of HPs (4/23) expressed a view that government regulation is not needed at this time, as there is no evidence that the self-regulated moratorium is inadequate. Several HPs cited the lack of discrimination witnessed by them personally since the moratorium started as the reason why further regulation was currently unnecessary.

“[Self-regulation] *has been working well, in that I’m not aware of any discriminatory cases that have come up … I guess the guidelines* [insurance industries] *are guided by with their current regulatory bodies have worked.”* (ID 11, certified genetic counsellor, 15-20 years’ experience)

#### Impact of the financial limits

Another significant concern reported by HPs is the effect on their patients of the moratorium’s financial limits. Of the 23 HPs interviewed, 18 discussed concerns with the financial limits, with 13 HPs expressing that such limits create a barrier for their patients in accessing testing.

*“So, whenever you raise the insurance question, I know* [patients say]*,’It’s only helpful up to half a million.’ And half a million isn’t as much as you used to think it was.”* (ID 14, certified genetic counsellor, >20 years’ experience)

HPs commented that patients often require polices worth more than $500,000, and that the current financial limit provides no reassurance to these patients.

*“There’s lots of people out there who mentioned that, if they were insuring their current income and there was a complete loss of income from this point onwards, that $500,000 [would not be sufficient] - they’d be looking at a much larger policy.”* (ID 15, clinical geneticist, 15–20 years’ experience)

Several HPs mentioned that the financial limit does not reflect the current cost of living. Particular references were made to the current property prices, and maintaining a mortgage and/or a personal business.

*“I’ve had a few people say it’s a bit low, this $500,000. Particularly I guess if you were someone who had your own business, or even a mortgage, and you wanted to make sure you were covered for that … So, it does seem a bit low when you consider what things cost.”* (ID 9, certified genetic counsellor, 11–15 years’ experience)

#### Uncertainty created by the temporary nature of the moratorium

Many HPs expressed concerns about the temporary nature of the moratorium, and how the uncertainty of its duration leaves HPs unable to advise or reassure clients with confidence. Many HPs mentioned that they are unable to provide patients with clarity around what will happen after the moratorium ends.

*“It’s very difficult to know because I think the term* [of the] *moratorium means that they’re building something that’s temporary … If someone gets insurance through the moratorium and then the moratorium ends, what does all of that mean?”* (ID 21, “other” HP, > 20 years’ experience)

Many HPs commented that they could not provide patients with any information or reassurance relating to how insurance companies will use patients’ information in the future.

“*We really don’t know what’s going to happen after 2024 … and nobody really knows what the impact’s going to be – what the insurance industry or anybody else is doing with that information in those years to come.”* (ID 19, clinical geneticist, >20 years’ experience)

The temporary nature of the moratorium featured in a number of HPs’ descriptions of the difficulty of explaining the moratorium to patients, and its effect on the reassurance they can provide to patients, as well as their own uncertainty about whether the moratorium will continue to apply in the future.

*“So some [counsellors] are saying, ‘So I don’t know what will happen after that, it might be wiped.’ And so I think for some, that’s a reason to say, ‘Look, this protection may not apply soon.’ So there’s a little uncertainty there, and a little less, I guess, reassurance that we can provide.” (ID 5, associate genetic counsellor, 0*–*5 years’ experience)*

Some HPs said that the temporary nature of the moratorium created further complexities, not only for their own understanding about how the moratorium is applied, but also difficulties explaining this limitation to patients.

*“It’s a short-term thing and it’s not entirely clear what it means. So I think that makes it difficult to explain.”* (ID 18, clinical geneticist, >20 years’ experience)

## Discussion

Our study provides an in-depth assessment of Australian HPs’ views and experiences regarding the current FSC moratorium on genetic testing and life insurance.

Interviews with 23 of the 166 previous participants of our published online survey [22], allowed us to obtain a more in-depth understanding of HPs’ views and experiences. Capturing these views and experiences is an important part of adequately informing future policy. The findings emerged within two major themes – HPs’ views on the benefits of the moratorium, and their concerns. Although some perceived benefits of the current moratorium were articulated by HPs, the major finding of our study was the consistent concerns raised, especially regarding the temporary nature of the moratorium (creating uncertainty for patients and HPs), the financial limits - which in the assessment of HPs are too low – and the issues with self-regulation by the insurance industry. The majority of interviewed HPs felt that the best solution to genetic discrimination in life insurance in Australia is government regulation or legislation.

While HPs generally consider that the moratorium is an important first step in reducing genetic discrimination in life insurance, most HPs expressed continuing concerns about the temporary nature of the moratorium, its financial limits and industry self-regulation. These concerns mirror those expressed by HPs in the previous online survey [22], where >90% of HPs expressed views that government regulation and legislation regarding the use of genetic test results in underwriting are required. In our qualitative follow-up interviews, Australian HPs again frequently highlighted the need for more stringent regulation, both to reassure patients and to ensure compliance by insurance companies.

### Temporary nature of the moratorium

Many HPs expressed discomfort with their inability to reassure patients due to the temporary nature of the moratorium. Despite a recommendation from the Parliamentary Joint Committee (PJC) in 2018 that any ban should apply indefinitely to genetic tests taken before the moratorium is lifted, to ensure certainty for consumers [13], this protection was not incorporated into the FSC moratorium. As anticipated by the Parliamentary Joint Committee, this uncertainty is now impacting patients and their HPs’ ability to provide them with adequate information. The temporary nature leaves HPs unsure of how the moratorium will be applied in the future, therefore increasing the complexity of the insurance and genetic testing conversations they have with patients. Furthermore, HPs cannot reassure patients that they will remain protected in the future, creating uncertainty for both patients and HPs.

HPs who are involved in organising genetic testing must provide patients with information regarding the medical and familial implications of a genetic condition, while working collaboratively to plan the next healthcare steps [26]. This role includes helping patients decide whether to have genetic testing. Obtaining informed consent for genetic testing requires HPs to provide information regarding the risks and benefits of undergoing such a test, which includes a discussion around insurance implications [27]. More specifically, in Australia the professional guidelines for genetic counsellors (who comprised ~60% of the online survey participants and 60% of our interview participants) require a discussion of the insurance implications to be included in consultations where relevant [28].

Our interview data demonstrates that many Australian HPs are now unsure how to have conversations about genetic testing and life insurance with patients, given the uncertainty around the future of the moratorium, and the possible future insurance implications of having genetic testing at this time. Given the possibility that the moratorium may not be continued beyond 2024, it is indeed impossible for HPs or any person to provide reliable or guaranteed information about the future insurance risks of genetic testing.

### Financial limits

Pre-moratorium, Australian life insurance companies could ask applicants about genetic test results regardless of the amount of cover being applied for. Under the partial current moratorium, patients can apply for life insurance policies up to $ 500,000 without disclosing genetic test results [16]. As indicated by some of the interviewed HPs, this has allowed patients access to a baseline level of insurance cover which was not previously possible. However, the majority of HPs indicated that a proportion of their patients perceived the current financial limits as a significant restriction, with some patients finding the limits too low to adequately cover their financial needs. According to the Australian Bureau of Statistics, as at December 2021 the average loan size for owner-occupier dwellings was $ 602,000 [29].

Concerns with the moratorium’s financial limits were similarly reflected in our previously published survey, where almost half of the responding HPs made comments in the optional comments section regarding the moratorium’s financial limits being too low [22]. These concerns were echoed in the qualitative HP interviews, highlighting HPs’ widespread concerns that the moratorium’s financial limits are inadequate to protect patients. Despite the FSC’s public statement announcing the moratorium that “the insurance cover limits compare favourably with other countries” [30], analysis shows that countries which have such financial limits, let alone still allow the use of genetic data in life insurance underwriting, are in the minority [7].

### Concerns regarding self-regulation

When exploring HPs’ views on the moratorium’s regulation, a majority of participants voiced the need for life insurers’ use of genetic test results to be regulated by government. This result was consistent with our previous online survey, in which 95% (*n* = 166) felt that government oversight of the moratorium is required [22]. In both analyses, we observed strong dissatisfaction with self-regulation by the insurance industry, paired with HP distrust in insurance companies’ compliance with the moratorium terms. While a minority of HPs considered that the self-regulated nature of the moratorium is an adequate solution to address genetic discrimination, the majority felt that government regulation is needed to ensure compliance by insurance companies, and to provide a long-term regulatory solution.

Self-regulation in the Australian financial services industry has been criticised repeatedly in recent years, including for lack of transparency and compliance without external regulation [12, 13]. In 2019, an Australian Royal Commission was conducted to assess the misconduct in the banking, superannuation and the financial services industries. The findings reflected various issues that are inherent with industry self-regulation, including conflicts of interest and the industry’s failure to monitor and enforce compliance with their codes [31].

For some HPs, the solution to the issue of non-compliance by the insurance industry is to move from self-regulation to government regulation. The introduction of legislation was expressed by many HPs as a necessary intervention to ensure compliance with regulations, and provide consequences for non-compliance. Our findings suggest that the majority of Australian HPs who discuss genetic testing with patients perceive industry self-regulation of the moratorium to be inadequate, and consider government regulation necessary.

Discussions about regulation in this area sometimes raise queries about consequences of restricting insurers’ access to genetic information. Some insurers and authors have raised concerns that the restriction on the use of genetic test results by insurers may lead to “adverse selection”, whereby the purchase of insurance by individuals with genetic predisposition to disease could lead to unsustainability of the insurance sector [32]. This issue has been raised internationally, including recently when the Canadian regulation on this issue was being considered. The Canadian Privacy Commissioner commissioned several actuarial experts to undertake modelling to assess the impact of a ban on using genetic test results in life insurance underwriting [33]. Each of these experts concluded that a ban would have negligible market impact at the time [34, 35], and were relied on by the Privacy Commissioner in assessing the appropriateness of regulation [36]. Canada eventually passed the *Genetic Non-Discrimination Act* in 2017, which bans the use of genetic test results in the provision of any goods or services (including insurance), with criminal penalties. This issue was also raised by the Australian life insurance industry in the recent Australian Parliamentary Joint Committee inquiry [13]. The Committee commented in its report that, “the committee notes the reasoning underlying the insurance industry’s need for genetic information. However, fears that adverse selection as a consequence of consumers not having to disclose predictive genetic testing results would make the life insurance market unsustainable may be overstated. In addition, the Canadian Office of the Privacy Commissioner found that the sustainability of the Canadian insurance industry is not likely to be affected at this time by a ban on the use of genetic information. Life insurers did not provide strong evidence to the contrary… Though the committee considers the fears overstated, the committee acknowledges adverse selection as a phenomenon in insurance. The committee’s primary concern in that regard is the potential for higher costs for consumers if information asymmetry between insurers and insureds causes insurers to seek to put up premiums to compensate. However, on balance, the committee believes there is presently greater benefit to consumers in preventing a duty of disclosure from arising in respect of predictive genetic tests for the reasons referred to above”.

Limitations of this study include the relatively small number of interviewed participants, and the potential for self-selection bias that may have influenced participation (e.g. HPs who volunteered to participate may be more likely to be engaged and have strong views about the topic, compared to other HPs). Nonetheless, we interviewed all HPs who consented to an interview, and continued interviewing participants until after data saturation was reached, to capture as many viewpoints as possible. A further limitation of our study is the secondary nature of reports by HPs about patient views and experiences. Some of our findings are therefore limited to HPs’ understanding and experience of patient views, rather than the collection of direct evidence. Past research by this group has demonstrated significant consumer concerns regarding the use of genetic test results by consumers existed before the implementation of the moratorium [37]. Further studies have been designed to gather updated views from Australian consumers and patients directly about this issue [17] and will be reported separately.

Strengths of the study include the sequential, explanatory mixed method design, which allows for both quantitative analysis of survey data (previously published) and in-depth exploration of the previous survey responses to obtain a more complete picture of HPs’ views and opinions.

In conclusion, our study demonstrates that although Australian HPs consider the FSC moratorium to be a positive step that provides benefits for some patients, ultimately the majority of HPs remain concerned about the overall adequacy of the current moratorium as a long-term regulatory solution. Major concerns raised with the moratorium include its industry self-regulation; low financial limits; and temporary nature, resulting in uncertainty of future applicability. Most HPs consider that government regulation and intervention is required to adequately protect Australian consumers long-term. The findings of this study indicate the need for a more stable, independent and long-term policy solution for the regulation of genetic testing and life insurance in Australia.

## Supplementary information

Supplementary File S1

Supplementary File S2

## Data Availability

Some data is made available via supplementary materials. Additional data can be made available on reasonable request.
