# Supplementary material for: Health professionals’ views and experiences of the Australian moratorium on genetic testing and life insurance: A qualitative study
Source: Eur J Hum Genet. 2022 Jul 28;30(11):1262–8. doi: 10.1038/s41431-022-01150-6 (PMC9626480; doi:10.1038/s41431-022-01150-6)
Supplement: Supplementary file 1 — Supplementary File S1 [file 41431_2022_1150_MOESM1_ESM.docx]

**Supplementary File S1: Generic interview schedule**

Before we get started, I wanted to confirm that you are aware that this interview will be recorded, and that the content provided here will be de-identified, analysed and used in my masters project, as well as future research.

Are you still happy to participate?

As you know you completed a survey about the moratorium on insurance and genetic testing. I have your survey answers here and I will refer to them and get you to expand on some of your response.

| **Broad themes** | **Main Questions** | **Follow up prompts (if not raised)** |
| --- | --- | --- |
| **Intro- Moratorium** | - As you know the moratorium has been introduced, what do you think of it so far? - How does your clinical experience compare after the introduction of the moratorium? - In the survey you expressed that you feel you have**/don’t have** sufficient knowledge on the moratorium to advise clients. Can you tell me why you feel this way?/ **What would help you feel more comfortable?** | - In what ways has the moratorium impacted your clients? (Has the moratorium reduced the number of people who refuse or delay testing?) - What are the benefits of the moratorium? - What are the limits of the moratorium? |
| **Regulation** | - In the survey you indicated that you are **very satisfied/somewhat satisfied/somewhat dissatisfied/very dissatisfied** with the use of the moratorium as a solution to genetic discrimination in life insurance. Can you tell me why this is? - In the survey you indicated that life insurers’ compliance with the moratorium should be regulated through **legally enforceable rules/ self-regulation by the life insurance industry.** Can you please expand on this? - You indicated that the Australian government **should/ should not** introduce legislation to regulate the use of genetic test results in life insurance. Can you tell me more about this? | - In your professional opinion what is the best solution to resolve genetic discrimination in life insurance? |
| **Personal practice with clients (where applicable)** | - Can you tell me how the moratorium has impacted your interaction with clients? - You indicated that clients are **more willing/ not willing**  to have genetic testing since the moratorium, are you able to expand on why this is? | - From the survey I can see you **agreed/disagreed** that the moratorium is easy for clients to understand. Can you tell me why it is easy/difficult for clients, and how you explain it to clients? - You indicated that you **agree/disagree** with the statement “the moratorium has resolved some concerns I had about insurance discrimination” can you tell me why this is the case? |
| **Clinic practice** | - You have indicated that your health service has a **written/verbal** policy regarding communication with patients about insurance implications of genetic testing.  Can you tell me if there has been a change to this since the moratorium? - You have indicated that your health service **does not** have a policy regarding communication with patients about insurance implications of genetic testing. What are your views on this? Do you think a policy is necessary? | - Do you think your health service should have a policy? |
| **Training and education offered by clinic** | - You’ve indicated that you **have** received training at your genetics service which was **not adequate.** Can you tell me a bit about what training you would find useful? - You’ve indicated that you **have not** received training at your genetics service. Can you tell me a bit about what training you would find useful? | - Are insurance issues frequently/ever discussed at team meetings/amongst colleagues? - What resources have been most helpful? |

END OF SESSION: That concludes my questions. Are there any further comments you would like to make on this issue?
