# Supplementary material for: Health professionals’ views and experiences of the Australian moratorium on genetic testing and life insurance: A qualitative study
Source: Eur J Hum Genet. 2022 Jul 28;30(11):1262–8. doi: 10.1038/s41431-022-01150-6 (PMC9626480; doi:10.1038/s41431-022-01150-6)
Supplement: Supplementary file 2 — Supplementary File S2 [file 41431_2022_1150_MOESM2_ESM.docx]

**Supplementary File S2: Coding framework**

| Themes | Subthemes |
| --- | --- |
| The benefits of the moratorium, as reported by patients or observed by health professionals | Alleviating patient concerns   - Patients relieved that family members won’t have adverse insurance outcomes. - Patients more comfortable with genetic testing.   Removed barriers for patients   - Patients no longer have to sort out insurance before undergoing genetic testing. - Provides access to low level of insurance. - Reduces the number of clients stopping genetic testing.   Moratorium making practice easier   - Easy to explain the moratorium in simple situations. - Moratorium gives HPs concrete information to tell clients. |
| Concerns about the moratorium, as reported by patients or observed by health professionals | Temporary solution   - Unsure what will happen after the end date. - Patients fear they will be asked about genetic testing by insurance companies in the future. - Patients worried that their children’s own insurance will be affected in the future. - The temporary nature still causes some patients to delay genetic testing. - The need to continue to collect data on the moratorium to assess its effectiveness.   Financial limits   - Step in the right direction as moratorium offers some cover under the financial limit. - Patients discuss that the financial limit does not provide adequate cover. - HP’s comment that financial limit does not account for the average wadge and lifestyle.   Oversights of the moratorium   - Too many parties interested in the moratorium so this solution is too simple. - Disconnect between insurance and treatable conditions. - Does not alleviate concerns for clients that care about insurance implications.   Patients lack of awareness   - Patients unaware of impact on insurance. - Patients unaware of insurance in superannuation. - Patients who are aware of the insurance implications of genetic testing may not come to counselling as they fear discrimination by insurance companies.   Patients lack of certainty   - Patients get confused about existing insurance policies when the moratorium is brought up. - Patients can get confused by the moratorium’s limitations, so they put off testing. - Patients are unsure of their rights regarding the moratorium. - HPs cannot be reassuring as moratorium changes.   Self-regulation   - Self-regulation is not right as insurance companies have a commercial interest. - Self-regulation is not a good solution as HP’s distrust insurance companies. - HP’s believe that self-regulation means that insurance companies don’t have to be transparent. - Government oversight is a better solution than that of self-regulation. - Self-regulation is more flexible for insurance companies than legislation. - HPs lack of trust in insurance companies to follow self-regulation. |
| Implications of the moratorium for health professionals’ practice | HPs’ role of reassuring patients   - Not being able to reassure patients makes counselling difficult.   Role of HPs in insurance discussions   - Not HP’s role to give insurance advice, only to bring up implications of genetic testing. - Insurance questions are better for those who work in the insurance industry rather than HPs. - Some HPs are unsure whose role it is to talk about insurance.   HPs lacking confidence and knowledge   - Lack knowledge and confidence in advice giving. - moratorium is still ambiguous even with fact sheet. |
